# Cyberbullying Victimization and Suicide Attempt Among Adolescents: A Cross-National Comparison

**DOI:** 10.3390/ijerph22030385

**Published:** 2025-03-06

**Authors:** Meytal Grimland, Yuko Mori, Sigita Lesinskiene, Liping Li, Say How Ong, Samir Kumar Praharaj, Tjhin Wiguna, Zahra Zamani, Emmi Heinonen, Sonja Gilbert, Anat Brunstein Klomek, Andre Sourander, the EACMHS Study Group

**Affiliations:** 1 INVEST Research Flagship Center, University of Turku, 20014 Turku, Finland; meytal.grimland@gmail.com (M.G.); yumori@utu.fi (Y.M.); emmi.m.heinonen@utu.fi (E.H.); sonyle@utu.fi (S.G.); andsou@utu.fi (A.S.); 2 Research Centre for Child Psychiatry, Department of Child Psychiatry, University of Turku, 20014 Turku, Finland; 3 Clinic of Psychiatry, Institute of Clinical Medicine, Faculty of Medicine, Vilnius University, LT-01131 Vilnius, Lithuania; sigita.lesinskiene@mf.vu.lt; 4 School of Public Health, Shantou University, Shantou 515041, China; lpli@stu.edu.cn; 5 Department of Developmental Psychiatry, Institute of Mental Health, Singapore 539747, Singapore; say_how_ong@imh.com.sg; 6 Department of Psychiatry, Kasturba Medical College, Manipal 576104, India; samirpsyche@yahoo.co.in; 7 Manipal Academy of Higher Education, Manipal 576104, India; 8 Department of Psychiatry, Faculty of Medicine Universitas Indonesia, Dr. Cipto Mangunkusumo General Hospital, Jakarta 10430, Indonesia; twiga00@yahoo.com; 9 Faculty of Medicine, Tehran University of Medical Sciences, Tehran 13399-73111, Iran; zamanizhm@gmail.com; 10 Baruch Ivcher School of Psychology, Reichman University, Herzliya 4610101, Israel; 11 Department of Child Psychiatry, Turku University Hospital, 20520 Turku, Finland

**Keywords:** bullying, cyberbullying, suicide attempt, adolescents, cross-national comparison

## Abstract

The widespread use of the Internet among teenagers has raised concerns about cyberbullying and its impact on adolescent well-being. This study examined the association between cyberbullying victimization and suicide attempts among adolescents in high-income and low/middle-income countries. Data from six countries (Singapore, China, Iran, Indonesia, India, and Lithuania) were collected as part of the Eurasian Child Mental Health Study. A total sample of 9892 adolescents aged 13–15 years old (51.9% girls) was analyzed. Generalized estimating equation models with school-wise clusters were conducted. The prevalence of suicide attempts was 4.8%, with higher rates among girls. Cyberbullying victimization only was reported by 5.4% of the participants, while traditional bullying victimization only was reported by 19.2%. The study found that being a victim of combined (both traditional and cyberbullying) had the highest odds of suicide attempt in both high-income and low/middle-income countries. Emotional symptoms were identified as a moderator, influencing the association between combined bullying victimization and suicide attempt. These findings highlight the urgent need for global efforts to prevent and intervene in cyberbullying and its detrimental effects on adolescent mental health. The study emphasizes the importance of examining regional risk factors and implementing targeted interventions to address this growing public health concern.

## 1. Introduction

The presence of the Internet in the social lives of teens worldwide has brought both hopes and fears regarding its effect on the well-being of youngsters. One of the detrimental outcomes many are concerned about is cyberbullying, an alarming phenomenon in the past few years with the widespread use of the Internet and the pervasive ownership of mobile phones. For example, 90% of children in the UK own a smartphone by the age of 11 [1], and the percentage of adolescents who are online has almost constantly increased from 24% to 46% in the last ten years [2]. Cyberbullying is commonly defined as a “willful and repeated harm, by a person or a group, inflicted using computers, cell phones, and other electronic devices, aimed against a victim who cannot easily defend him or herself” [3].

Numerous studies have shown the adverse effect cyberbullying has on teens’ lives such as internalizing, externalizing, psychosomatic, and substance use problems [4,5,6,7,8]. A meta-analysis examining cyberbullying involvement and suicidal behavior showed a significant association between cyberbullying victimization and self-harm, suicidal ideation, and suicide attempt [9]. The authors conducted a sensitivity analysis investigating traditional bullying victimization and suicidal behavior and found similar results. They concluded that being exposed to cyberbullying victimization puts youngsters at risk for suicidal behavior above and beyond traditional bullying victimization. Of all biological, genetic, perinatal, and ecological risk factors, the most alarming one regards suicide attempt since it is one of the strongest predictors of death by suicide [10]. Given that suicide is the fourth leading cause of death among 15–19-year-olds for both sexes worldwide [11], global efforts are needed for prevention and early intervention.

Most of the previous studies reporting an association between cyberbullying and suicide attempt are from high-income countries (HICs) [12]. Research data from low- and middle-income countries (LMICs) are scarce, possibly due to limited research infrastructure, publication in non-English journals, and reliance on gray literature. Studies from LMICs are crucial since more than half of the globe’s population is inhabited in these areas, and nearly 90% of the world’s adolescents live there. Moreover, more than 77% of the world’s suicides occur in LMICs. Similarly, most adolescents who died of suicide (88%) were from low- and middle-income countries [11]. Some of these studies published data of a single LMIC [13,14,15,16], but very few published data from multiple countries [17]. Cross-national studies, which use comparable questionnaires in all countries involved, narrow down variations in sampling and survey methods. Beyond that, examining cyberbullying victimization and suicidal behavior cross-nationally enables a better understanding of regional risk factors and, henceforth, targeted interventions. For example, the work of Nock and his colleagues [18] showed different predictors for suicidal attempts in HICs (mood disorder) compared to LMICs (impulse control disorders). This finding suggests that public health efforts should be tailored according to particular mental health problems emerging from country-specific data.

So far, cross-national studies that included LMICs have mostly been based on the Global School-based Student Health Survey (GSHS). This cross-sectional survey focuses on health behaviors and risk factors among adolescents attending school. All published works based on GSHS have focused on the correlation between traditional bullying victimization and suicide attempts. For example, Koyanagi and his colleagues [19] used data from 48 countries, from different continents, mostly from LMICs. All but one country showed that traditional victimization was significantly associated with more than 2-fold higher odds for a suicide attempt, suggesting this may be a global health concern. Campisi and her colleagues [20] used up-to-date data of the GHSH with almost double the number of countries and similarly showed an association between traditional bullying victimization and suicide attempt. It did not differ by age or gender. Tang and his colleagues [21] showed associations between traditional bullying victimization and suicidal ideation, suicide planning, and suicide attempt, with substantial variations across countries. Notably, the association between traditional bullying victimization and suicide attempt was more robust than traditional bullying and suicidal ideation/suicide plan. One cross-national study examined the association between cyberbullying victimization and suicidal behavior among 15-year-olds in high-income countries (Israel, Lithuania, and Luxembourg) based on data from Health Behaviour in School-aged Children (HBSC), a collaborative cross-national study organized by World Health Organization [17]. The study found that 6.5% of adolescents reported cyberbullying victimization, 9.5% attempted suicide in the past year, and those who were cyberbullied had a significantly higher risk of suicide attempt.

Reviewing the current knowledge, it appears that there is a lack of cross-national studies examining the association between cyberbullying victimization and suicide attempt both in LMICs and HICs. Previous studies investigating GSHS and HBSC data did not analyze covariates such as emotional symptoms, including depressive symptoms and anxiety, that have been found to be moderating bullying victimization and suicidal behavior [22,23]. Although the previous research has largely focused on emotional symptoms as a mediator in the association between bullying victimization and suicidal behavior, it has been suggested that it is also important to investigate the role of emotional symptoms as a moderator in this association [24].

The aim of the current study is to explore cyberbullying victimization and suicide attempt cross-nationally. More specifically, the first aim of the current study is to examine the prevalence and associations between cyberbullying victimization and suicide attempt in HICs and LMICs. The second aim is to explore the moderating effect of emotional symptoms on the association between bullying victimization and suicide attempt. According to the literature, we put forward the first hypothesis that the association between cyberbullying victimization and suicide attempt will differ in HICs and LMICs. Our second hypothesis is that emotional symptoms moderate the association between cyberbullying victimization and suicide attempt.

## 2. Materials and Methods

### 2.1. Sample

This study is part of the Eurasian Child Mental Health Study (EACMHS). EACMHS is an international cross-sectional study that aims to collect data on children and adolescents’ well-being and mental health [25]. The current study included data from five Asian countries and one European country: Singapore, China, Iran, Indonesia, India, and Lithuania, respectively. The country selection was based on whether they collected comparable data regarding suicide attempts. The survey was conducted between 2014 and 2016 for a total sample of 14,677 adolescents. Since there were variations in the age ranges in the total samples across countries, only 13–15-year-old adolescents were included in this study to increase the comparability of the data (mean age: 13.9, standard deviation: 0.8). We included a subsample of 9892 adolescents with available gender and suicide attempt data from 110 schools. The individual country sample sizes ranged from 545 in Iran to 2453 in Lithuania, with a mean of 1559. This sample consisted of 51.9% girls and 48.1% boys. The number of schools joining the study across the countries ranged from 5 in Indonesia to 44 in Lithuania, with a mean of 18. The survey year and the characteristics of the study sample in each country are presented in Table 1.

### 2.2. Questionnaire and Procedure

This study was conducted using a self-administered questionnaire based on previous work performed in Finland [26,27]. The questionnaires were translated into the local language and back-translated in each country to ensure accuracy. All students present in the class at the time of the survey were invited to participate. The questionnaires were administered by the teachers, and students completed the questionnaires during school classes. Teachers then collected the questionnaires in an enclosed envelope and handed them to the researchers. An electronic version of the questionnaire was used in Singapore. Ethical approval for the study was obtained from the institutional review boards in each participating country, with additional permissions obtained from the participating schools. Participation was entirely voluntary, and both anonymity and confidentiality of participants were ensured. The researchers obtained consent from the parents or school authorities according to each country’s policies at the time of the study. This study was performed in line with the principles of the Declaration of Helsinki and its later amendments.

### 2.3. Measures

#### 2.3.1. Bullying Victimization

Traditional bullying was defined as “A student is getting bullied, if another student or a group of students repeatedly treats him/her negatively or in an insulting manner. It is difficult for the bullied student to defend himself/herself. Bullying can be intermittent or continuous. Bullying can be verbal (e.g., calling names, threatening), physical (e.g., hitting, pushing), or psychological (e.g., spreading rumors, avoiding, excluding). Continuous nasty or insulting teasing is also bullying”. Students were then asked how often they had been bullied at school or outside of school in the past six months. We combined the responses into binary outcomes: no for never and yes for all other options. Cyberbullying was defined as “when someone repeatedly makes fun of another person online or repeatedly picks another person through email or text messages or when someone posts something online about another person that they do not like”. Students were then asked: “During the past six months, how often have you been cyberbullied?” Answers ranged on a 4-point Likert scale from 0 (“never”) to 3 (“almost every day”). The responses were similar to traditional bullying victimization and were combined into a binary outcome.

#### 2.3.2. Suicide Attempt

Suicide attempt was measured using one item, “Have you tried to commit suicide?” The responses varied between countries; they were binary (Iran, India, Singapore) or on a 3-point scale (China, Lithuania). For 3-point Likert-type response options, we combined the responses into binary outcomes: “no” for no or never, and “yes” for the other options.

#### 2.3.3. Covariates and Moderators

The covariates included demographics: age and sex. The moderator variable emotional symptoms were measured using the Strengths and Difficulties Questionnaire (SDQ) [28]. Answers ranged between 0 (“not true”) and 2 (“certainly true”). The SDQ is a widely used questionnaire with five subscales, each of which contains five items measuring emotional symptoms, conduct problems, hyperactivity, peer problems, and prosocial subscale [28]. The validity and reliability of the SDQ for self-completion of 11- to 17-year-old children or adolescents have been found to be satisfactory [29,30]. The SDQ has been used in previous cross-national studies [31,32].

### 2.4. Statistical Analysis

Descriptive analysis was made to depict the demographic characteristics and prevalence of reported suicide attempt and bullying victimization in the six countries separately and in the total sample. Generalized estimating equation (GEE) models with school-wise clusters were conducted to compare prevalence of suicide attempts between different types of bullying victimization in each country. Adjustments were made for the gender and age of the participants. Associations were reported as odds ratios (ORs) with 95% confidence intervals (95% CIs). To explore the moderating role of emotional symptoms, a GEE model with the interaction term adjusted by gender, age, and country was conducted. Indonesia was excluded from these models because there were no girls who had been cyberbullied only during the previous six months among those who had attempted suicide. All statistical analyses were performed using SAS 9.4 for Windows (SAS Institute Inc., Cary, NC, USA, 2012).

## 3. Results

The prevalence of reported suicide attempts and bullying victimization is shown in Table 2. No significant interactions between gender and explanatory variables on suicide attempts were found and henceforth none reported. The overall prevalence of suicide attempts was 4.8% (girls, 6.3%; boys, 3.2%) and ranged from 2.2% in Indonesia to 8.1% in Iran. The overall prevalence of past 6 months for cyber victimization only was 5.4% (range 1.3% in India to 6.5% in Indonesia; girls, 5.2%; boys, 5.7%) and the prevalence for traditional bullying victimization only was 19.2% (range 12.8% in China to 26.2% in Lithuania; girls, 17.2%; boys, 21.3%). The prevalence for combined victimization was 6.8% (range 2.1% in India to 13.8% in Indonesia; girls, 6.9%; boys, 6.7%). The prevalence of reported bullying victimization among those adolescents with suicide attempts is shown by country in Table S1 in the online Supplementary Material.

**Figure 1:**
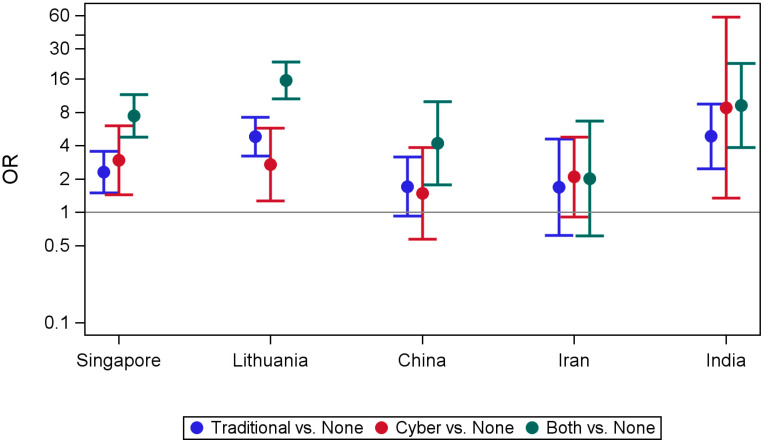
Association between suicide attempt and bullying victimization by country estimated by GEE models adjusted for age and gender. Both refer to both traditional victimization and cyberbullying victimization.

Table 3 presents the result of univariate and multivariate analyses examining the association between suicide attempt and bullying victimization by country. Figure 1 illustrates the results of the multivariate analysis adjusted for the age and gender of participants. In India, Lithuania, and Singapore, all three categories of victimization (traditional only, cyber only, combined) were associated with significantly higher odds for suicide attempt and the highest odds were in Lithuania for combined victimization (OR 15.61, 95% CI 10.61–22.94). In China, combined victimization was significantly associated with suicide attempt (OR 4.22, 95% CI 1.77–10.02) but not traditional victimization only and cyber victimization only. There was no significant association in Iran with all three categories of victimization.

When different victimization groups were compared, adolescents who were victims of combined bullying had the highest odds for suicide attempt compared with those who were not bullied, except Iran, which had no significant association. Of note, both Singapore and Lithuania, countries indexed as the highest income in our sample, combined victimization had significantly the highest odds for suicide attempt compared to traditional bullying victimization only. In India and Singapore, those who were cyberbullied only had the second highest odds for suicide attempt compared with those who were not bullied (OR 8.83, 95% CI 1.34–58.03; OR 2.95, 95% CI 1.44–6.03, respectively). In Lithuania, those who were traditionally bullied only had the second highest odds for suicide attempt compared with those who were not bullied (OR 4.85, 95% CI 3.23–7.27).

**Figure 2:**
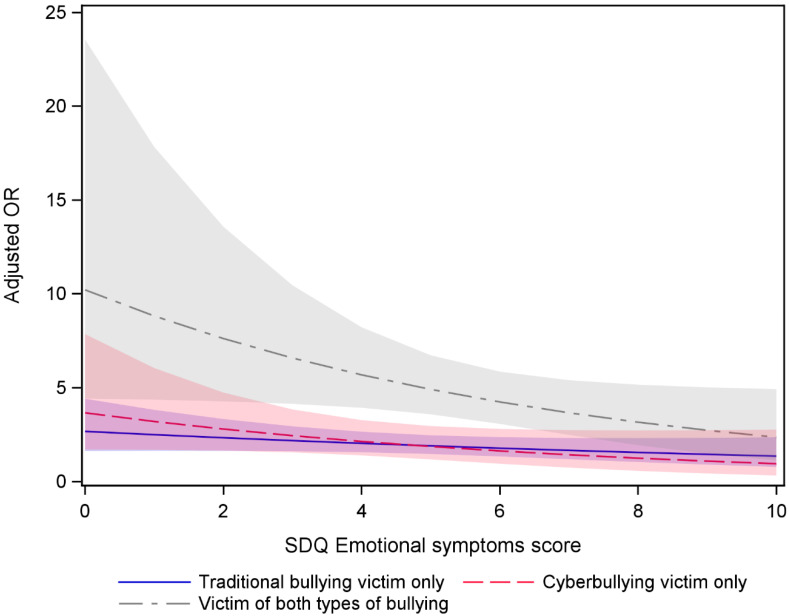
Adjusted Odds Ratio of suicide attempt and their 95% confidence intervals by victimization type and emotional score adjusted for country, age, and gender. Colored shadows represent confidence interval for each victimization type. The reference category was no bullying victimization.

In examining the possible moderator role of emotional symptoms, results indicated that emotional symptoms moderated the association between suicide attempt and victimization (Figure 2). The result revealed that emotional symptoms moderate the association between combined bullying victimization (both traditional and cyberbullying) and suicide attempt (*p* < 0.05). The relationship between combined bullying victimization and suicide attempt differs according to the level of emotional symptoms. Higher levels of emotional symptoms increase the odds of a suicide attempt less among those who have experienced combined bullying compared to those who have not been bullied. For traditional victimization only and cyberbullying victimization only, the interactions were not significant.

## 4. Discussion

The present study examined the association between cyberbullying victimization and suicide attempt among adolescents and the moderating effect of emotional symptoms on the association in six countries (Singapore, China, Iran, Indonesia, India, and Lithuania). The findings were based on data from a school-based survey conducted between 2014 and 2016. Our main results are as follows: in India, Lithuania, and Singapore, all forms of victimization increased suicide attempt odds, while China showed an association only for combined victimization, and Iran showed no significant association. Being a victim of both traditional and cyber victimization had the highest odds of suicide attempt compared with those who were not bullied in both HICs and LMICs, except Iran with no significant association. Our results also indicate a moderating effect of emotional symptoms on the association between combined bullying victimization and suicide attempt, but interactions were not significant for traditional bullying victimization only and cyberbullying victimization only.

The overall prevalence of suicide attempts was 4.8% (6.3% for girls and 3.2% for boys). The higher prevalence rate of girls’ suicide attempt is consistent with what is known in the literature as the gender paradox [33]. However, a rather recent study, which gathered data of 13–17-year-olds, from 90 countries, including many LMICs, showed no difference in the suicide attempt rate between boys and girls [20]. These findings emphasize the need for research on suicide behavior in LMICs, as most of what is known of the gender paradox was predominantly established in HICs.

The overall prevalence of past 6 months for cyber victimization only was 5.4% (range 1.3% in India to 6.5% in Indonesia; girls, 5.2%; boys, 5.7%) and the prevalence for traditional bullying victimization only was 19.2% (range 12.8% in China to 26.2% in Lithuania; girls, 17.2%; boys, 21.3%). The prevalence for combined victimization was 6.8% (range 2.1% in India to 13.8% in Indonesia; girls, 6.9%; boys, 6.7%). The results show that traditional victimization is the most prevalent form of bullying, similarly to other studies [34]. For example, the Global School-based Student Health Survey (GSHS) reported a prevalence of 4% of cyberbullying victimization. They also showed a wide variation in the prevalence of cyberbullying across countries and gender [35]. The reported prevalence rate of traditional bullying victimization in GSHS studies was around 30% [19,36,37], a slightly higher rate than in our findings. Differences may be explained by the variations in research methods and the countries included in the studies. The prevalence rate of combined victimization (cyber and traditional) is similar to another published paper of the EACMH study, the work of Chudal et al. [38], which included more countries than this study, strengthening our finding. Beyond Europe and Asia, a large US study also implied a higher combined victimization rate, with more than half of those who reported cyber victimization also reported various forms of traditional bullying victimization (relational, physical, and verbal) [39].

One of our most important findings was that combined victimization was associated with suicide attempt in all countries but one. Adolescents who were victims of combined victimization had the highest odds of suicide attempt compared with those who were not bullied, though the odds differed across countries (range 4.22 in China to 15.61 in Lithuania), except in Iran with no significant association. In three of the six countries, cyber victimization was significantly associated with suicide attempt (range 2.71 in Lithuania to 8.83 in India). This implies that not only does traditional bullying victimization hold a risk for suicide attempts in LMICs, as previously shown in the literature [19,21], but also that cyber victimization, and moreover, combined victimization, holds a risk too. It seems that there is a dose–response effect; the more an individual suffers from victimization, the greater suicide risk they are at in all countries included except Iran. However, our findings suggest the odds are the highest in HICs (Singapore and Lithuania), and the work of Hinduja and Patchin [39] from the United States echoes this finding. They showed that those who experienced both forms of bullying victimization were more than 11 times as likely to attempt suicide than those who were not exposed to bullying victimization.

We also explored the possible role of emotional symptoms. Our results indicated that emotional symptoms moderate the association between combined bullying (both traditional and cyberbullying) victimization and suicide attempt. The result implies that the impact of combined bullying on suicidal attempt is significantly influenced by the presence of pre-existing emotional symptoms. This finding is in line with previous research from high-income countries showing the moderating effect of emotional symptoms on the association between bullying victimization and suicide attempt [22,23]. Moreover, those at higher risk for suicide attempt, across emotional scores, were again victims of combined victimization, followed by traditional victimization and then cyber victimization. Namely, our findings suggest it is the victimization in general that puts one at risk, not specifically cyber victimization. Li [40] portrayed cyberbullying as a “new bottle but an old wine”, meaning a form of traditional bullying, like physical, social, or verbal bullying. Supporting this, a positive association has been established between traditional victims and cyber victims both in HICs and LMICs [8,38].

The high prevalence of bullying victimization and suicide attempts observed in some countries highlights the critical need for routine screening for bullying and suicide behavior. The prevalence of suicide attempts was almost four times higher in Iran compared to Indonesia. In Singapore and India, cyber victimization had a higher risk for suicide attempt than traditional bullying, while traditional bullying had a higher risk in Lithuania. These cross-national differences provide insights into how the manifestation of risk factors may differ significantly across countries. It is important to recognize these differences to implement effective interventions and allocate resources where they are most needed. The impact of combined bullying victimization on suicidal attempts is significantly influenced by the presence of pre-existing emotional symptoms. Interventions aimed at reducing suicide rates among bullied adolescents should also focus on identifying and treating underlying emotional disorders. Future research should investigate causal links between bullying, emotional symptoms, and suicide attempts over time, and evaluate the effectiveness of interventions in reducing bullying behavior and suicide risks, especially in LMICs where data are limited. The study holds a few limitations; given the correlative nature of the cross-sectional study, causation cannot be inferred, and the results should be interpreted with caution. Cross-national longitudinal studies will be able to establish a temporal sequence in HICs and LMICs. There is some concern for social desirability, especially regarding mental health, whenever data were collected via self-reports [41]; using validated instruments [42] may facilitate overcoming the tendency to present oneself more favorably. More importantly, the study was conducted in certain geographical areas of those countries using a convenience sampling method. The aim was to select schools representing the diversity of the education system in each participating country, considering factors like urban/rural distribution and socioeconomic status. However, the reported prevalence rate refers to prevalence in those certain regions that participated in the study and may not represent the whole country. Although we aimed to include public and private schools from both urban and rural locations, the study sample largely consisted of public schools in countries like Singapore and Lithuania. This discrepancy was partially due to the different educational systems in countries. Given that the data were collected between 2014 and 2016, careful interpretation of the study results is needed, as the findings may not fully reflect current trends or contexts. A larger sample, with more students, in more schools, and in more countries, will better represent and henceforth enable a better understanding of the complex association between cyberbullying victimization and suicide attempt. Despite these limitations, this study does address a gap in the literature; it is to the best of our knowledge the first to examine and compare the correlation between cyberbullying victimization and suicide attempts in HICs and LMICs.

## 5. Conclusions

The present study highlights an important cross-national difference in the prevalence and association of bullying victimization and suicide attempts among 13- to 15-year-old adolescents in six countries. Our findings reveal that the association between cyberbullying victimization and suicide attempts varied, with a significant association found in India, Lithuania, and Singapore, but not in China and Iran. Additionally, the moderating effect of emotional symptoms was observed for combined bullying victimization but not for traditional or cyberbullying victimization alone. Overall, the results suggest that bullying victimization, particularly when both traditional and cyberbullying are involved, significantly increases the risk of suicide attempts among adolescents. As bully victims of combined bullying are the most vulnerable group, and interventions targeting these individuals could help mitigate risk factors and reduce negative outcomes. Future research should focus on exploring the longitudinal effects of bullying on suicidal behaviors to gain a deeper understanding of the temporal dynamics of this association.

## Figures and Tables

**Table 1:** Description of the study sample from the Eurasian Child Mental Health Study.

Country	Income Level	Survey Year	Total Sample	Sub Sample	Girls ^a^	Rural Residence	Urban Residence	Public School	Private School	Age	Response Rate	Schools
			*n*	*n*	*n* (%)	*n* (%)	*n* (%)	*n* (%)	*n* (%)	Mean (SD)	%	*n*
Singapore	H	2014	3319	2157	1101 (51.0)	0 (0)	2157 (100)	2157 (100)	0 (0)	14.0 (0.8)	85.8	24
Lithuania	H	2016	3837	2453	1242 (50.6)	1131 (46.1)	1322 (53.9)	2453 (100)	0 (0)	14.1 (0.8)	81.0	44
China	UM	2016	2659	2090	1030 (49.3)	1284 (61.4)	806 (38.6)	1645 (78.7)	445 (21.3)	13.8 (0.8)	96.1	10
Iran	UM	2016	1456	545	**365 (67.0)**	0 (0)	545 (100)	429 (78.7)	116 (21.3)	14.1 (0.8)	97.1	16
Indonesia	LM	2016	1390	1020	541 (53.0)	0 (0)	1020 (100)	655 (64.2)	365 (35.8)	13.5 (0.6)	51.7	5
India	LM	2016	2016	1627	850 (52.2)	246 (15.1)	1381 (84.9)	202 (12.4)	1425 (87.6)	13.6 (0.7)	93.9	11
Total	LM-H	2014–2016	14,677	9892	**5666 (51.9)**	2661 (24.4)	8250 (75.6)	8560 (78.5)	2351 (21.6)	13.9 (0.8)	51.7–97.1	110

^a^ The Chi-square test for equal proportions was used to analyze gender distribution. Bold type indicates the statistical significance of at least *p* < 0.05. SD—standard deviation; H—high income; UM—upper middle income; LM—lower middle income; Income level classification was based on the World Bank classification at the year of the survey in each country.

**Table 2:** Prevalence of reported bullying victimization in the past six months and suicide attempt by country.

Country	Suicide Attempt (Yes)			Bullying Victimization											
	Overall	Girls	Boys	Overall				Girls				Boys			
				None	Traditional Only	Cyber Only	Combined	None	Traditional Only	Cyber Only	Combined	None	Traditional Only	Cyber Only	Combined
	*n* (%)	*n* (%)	*n* (%)	*n* (%)	*n* (%)	*n* (%)	*n* (%)	*n* (%)	*n* (%)	*n* (%)	*n* (%)	*n* (%)	*n* (%)	*n* (%)	*n* (%)
Singapore	131 (6.1)	97 (8.8)	34 (3.2)	1533 (71.2)	344 (16.0)	83 (3.9)	192 (8.9)	793 (72.1)	143 (13.0)	44 (4.0)	120 (10.9)	740 (70.3)	201 (19.1)	39 (3.7)	72 (6.8)
Lithuania	143 (5.8)	87 (7.0)	56 (4.6)	1456 (62.4)	610 (26.2)	109 (4.7)	157 (6.7)	748 (62.4)	312 (26.0)	64 (5.3)	75 (6.3)	708 (62.5)	298 (26.3)	45 (4.0)	82 (7.2)
China	79 (3.8)	55 (5.3)	24 (2.3)	1581 (78.0)	260 (12.8)	116 (5.7)	70 (3.5)	819 (81.5)	107 (10.7)	49 (4.9)	30 (3.0)	762 (74.6)	153 (15.0)	67 (6.6)	40 (3.9)
Iran	44 (8.1)	33 (9.0)	11 (6.1)	345 (65.5)	90 (17.1)	57 (10.8)	35 (6.6)	243 (70.0)	49 (14.1)	41 (11.8)	14 (4.0)	102 (56.7)	41 (22.8)	16 (8.9)	21 (11.7)
Indonesia	22 (2.2)	11 (2.0)	11 (2.3)	572 (56.1)	241 (23.6)	66 (6.5)	141 (13.8)	312 (57.7)	111 (20.5)	37 (6.8)	81 (15.0)	260 (54.3)	130 (24.1)	29 (6.1)	60 (12.5)
India	42 (2.6)	23 (2.7)	19 (2.5)	1155 (76.7)	300 (19.9)	19 (1.3)	32 (2.1)	650 (82.5)	122 (15.5)	7 (0.9)	9 (1.1)	505 (70.3)	178 (24.8)	12 (1.7)	23 (3.2)
Total	525 (4.8)	355 (6.3)	170 (3.2)	7248 (68.6)	2024 (19.2)	573 (5.4)	717 (6.8)	3899 (70.7)	949 (17.2)	285 (5.2)	381 (6.9)	3349 (66.3)	1075 (21.3)	288 (5.7)	336 (6.7)

Combined refers to both traditional victimization and cyberbullying victimization.

**Table 3:** Univariate and multivariate analyses of the association between suicide attempts and bullying victimization.

Country	Bullying Victimization	Univariate Analyses			Multivariate Analyses ^a^		
		OR	95% Cl	*p* Value	OR	95% Cl	*p* Value
Singapore	Traditional only	**2.07**	**(1.32–3.24)**	0.0014	**2.32**	**(1.50–3.58)**	0.0001
	Cyber only	**2.92**	**(1.45–5.86)**	0.0027	**2.95**	**(1.44–6.03)**	0.0031
	Combined	**7.90**	**(5.17–12.07)**	<0.0001	**7.47**	**(4.80–11.63)**	<0.0001
Lithuania	Traditional only	**4.67**	**(3.10–7.03)**	<0.0001	**4.85**	**(3.23–7.27)**	<0.0001
	Cyber only	**3.06**	**(1.39–6.77)**	0.0056	**2.71**	**(1.27–5.79)**	0.0102
	Combined	**14.19**	**(9.17–21.98)**	<0.0001	**15.61**	**(10.61–22.94)**	<0.0001
China	Traditional only	1.54	(0.86–2.75)	0.1478	1.71	(0.92–3.16)	0.0884
	Cyber only	1.33	(0.53–3.34)	0.5446	1.48	(0.57–3.84)	0.4200
	Combined	**3.83**	**(1.63–8.98)**	0.0021	**4.22**	**(1.77–10.02)**	0.0011
Iran	Traditional only	1.33	(0.49–3.60)	0.5731	1.69	(0.62–4.62)	0.3077
	Cyber only	2.42	(1.00–5.88)	0.0509	2.09	(0.91–4.80)	0.0815
	Combined	1.88	(0.47–7.47)	0.3723	2.02	(0.61–6.66)	0.2466
India	Traditional only	**4.52**	**(2.06–9.89)**	0.0002	**4.87**	**(2.47–9.57)**	<0.0001
	Cyber only	**8.96**	**(1.33–60.22)**	0.0241	**8.83**	**(1.34–58.03)**	0.0233
	Combined	**7.95**	**(2.87–21.98)**	<0.0001	**9.25**	**(3.85–22.25)**	<0.0001

Bold type indicates statistical significance of at least *p* < 0.05. ^a^ adjusted for age and sex. OR Odds Ratio. CI Confidence interval.

## Data Availability

The data that support the findings of this study are not openly available due to reasons of sensitivity and are available from the corresponding author upon reasonable request.

## Supplementary Materials

The following supporting information can be downloaded at: https://www.mdpi.com/article/10.3390/ijerph22030385/s1, Table S1: Prevalence of reported bullying victimization in the past 6 months in those adolescents with suicide attempt by country.
