# Supplementary material for: Cyberbullying Victimization and Suicide Attempt Among Adolescents: A Cross-National Comparison
Source: Int J Environ Res Public Health. 2025 Mar 6;22(3):385. doi: 10.3390/ijerph22030385 (PMC11941981; doi:10.3390/ijerph22030385)
Supplement: Supplementary file 1 [file ijerph-22-00385-s001.zip › ijerph-3393445-supplementary.pdf]

Title: Cyberbullying Victimization and Suicide Attempt among Adolescents: A Cross-national Comparison

Meytal Grimland[1,3], Yuko Mori[2,3], Sigita Lesinskiene[4], Liping Li[5], Say How Ong[6], Samir Kumar Praharaj[7,8], Tjhin Wiguna[9], Zahra Zamani[10], Emmi Heinonen[2], Sonja Gilbert[3], Anat Brunstein Klome[11], Andre Sourander[2,12*], the EACMHS Study Group

[1]Shalvata Mental Health Center, Hod Hasharon, Israel

[2]Research Centre for Child Psychiatry, Department of Child Psychiatry, University of Turku, Turku, Finland

[3]INVEST Research Flagship Center, University of Turku, Turku, Finland

[4]Vilnius University, Faculty of Medicine, Institute of Clinical Medicine, Clinic of Psychiatry, Vilnius, Lithuania

[5]Shantou University Medical College, Shantou, China

[6]Department of Developmental Psychiatry, Institute of Mental Health, Singapore

[7]Department of Psychiatry, Kasturba Medical College, Manipal, India

[8]Manipal Academy of Higher Education, Manipal, India

[9]Department of Psychiatry, Faculty of Medicine Universitas Indonesia, dr. Cipto Mangunkusumo General Hospital, Jakarta, Indonesia

[10]Tehran University of Medical Sciences, Tehran, Iran

[11]Baruch Ivcher School of Psychology, Reichman University, Herzliya, Israel

[12]Department of Child Psychiatry, Turku University Hospital, Turku, Finland

*The corresponding author: Anat Brunstein Klomek (bkanat@runi.ac.il)

Table S1 Prevalence of reported bullying victimization in the past 6 months in those adolescents with suicide attempt by country

**Country**	**Bullying victimization**											
	**Overall**				**Girls**				**Boys**			
	None	Traditional only	Cyber only	Combined	None	Traditional only	Cyber only	Combined	None	Traditional only	Cyber only	Combined
	n (%)	*n* (%)	*n* (%)	*n* (%)	*n* (%)	*n* (%)	*n* (%)	*n* (%)	*n* (%)	*n* (%)	*n* (%)	*n* (%)
**Singapore**	54 (41.9)	24 (18.6)	8 (6.2)	43 (33.3)	41 (42.3)	16 (16.5)	4 (4.1)	36 (37.1)	13 (40.6)	8 (25.0)	4 (12.5)	7 (21.9)
**Lithuania**	32 (23.7)	58 (43.0)	7 (5.2)	38 (28.2)	26 (30.2)	34 (39.5)	4 (4.7)	22 (25.6)	6 (12.2)	24 (49.0)	3 (6.1)	16 (32.7)
**China**	52 (66.7)	13 (16.7)	5 (6.4)	8 (10.3)	40 (74.1)	8 (14.8)	3 (5.6)	3 (5.6)	12 (50.0)	5 (20.8)	2 (8.3)	5 (20.8)
**Iran**	23 (53.5)	8 (18.6)	8 (18.6)	4 (9.3)	17 (53.1)	6 (18.8)	7 (21.9)	2 (6.3)	6 (54.6)	2 (18.2)	1 (9.1)	2 (18.2)
**Indonesia**	6 (27.3)	10 (45.5)	1 (4.6)	5 (22.7)	2 (18.2)	6 (54.6)	0 (0.0)	3 (27.3)	4 (36.4)	4 (36.4)	1 (9.1)	2 (18.2)
**India**	16 (42.1)	17 (44.7)	2 (5.3)	3 (7.9)	11 (52.4)	8 (38.1)	1 (4.8)	1 (4.8)	5 (29.4)	9 (52.9)	1 (5.9)	2 (11.8)
**Total**	211 (41.6)	148 (29.2)	36 (7.1)	112 (22.1)	159 (45.6)	92 (26.4)	23 (6.6)	75 (21.5)	52 (32.9)	56 (35.4)	13 (8.2)	37 (23.4)

Combined refers to both traditional victimization and cyberbullying victimization.
